# Evolutionary transitions in the Asteraceae coincide with marked shifts in transposable element abundance

**DOI:** 10.1186/s12864-015-1830-8

**Published:** 2015-08-20

**Authors:** S. Evan Staton, John M. Burke

**Affiliations:** Department of Genetics, University of Georgia, Athens, GA 30602 USA; Current address: Beaty Biodiversity Research Centre and Department of Botany, 3529–6270 University Blvd, University of British Columbia, Vancouver, BC V6T 1Z4 Canada; Department of Plant Biology, University of Georgia, Athens, GA 30602 USA

## Abstract

**Background:**

The transposable element (TE) content of the genomes of plant species varies from near zero in the genome of *Utricularia gibba* to more than 80 % in many species. It is not well understood whether this variation in genome composition results from common mechanisms or stochastic variation. The major obstacles to investigating mechanisms of TE evolution have been a lack of comparative genomic data sets and efficient computational methods for measuring differences in TE composition between species. In this study, we describe patterns of TE evolution in 14 species in the flowering plant family Asteraceae and 1 outgroup species in the Calyceraceae to investigate phylogenetic patterns of TE dynamics in this important group of plants.

**Results:**

Our findings indicate that TE families in the Asteraceae exhibit distinct patterns of non-neutral evolution, and that there has been a directional increase in copy number of *Gypsy* retrotransposons since the origin of the Asteraceae. Specifically, there is marked increase in *Gypsy* abundance at the origin of the Asteraceae and at the base of the tribe Heliantheae. This latter shift in genome composition has had a significant impact on the diversity and abundance distribution of TEs in a lineage-specific manner.

**Conclusions:**

We show that the TE-driven expansion of plant genomes can be facilitated by just a few TE families, and is likely accompanied by the modification and/or replacement of the TE community. Importantly, large shifts in TE composition may be correlated with major of phylogenetic transitions.

**Electronic supplementary material:**

The online version of this article (doi:10.1186/s12864-015-1830-8) contains supplementary material, which is available to authorized users.

## Background

A common feature of eukaryotic genomes is that they contain transposable elements (TEs), yet there is a remarkable amount of variation in TE content and composition between species [1, 2]. This property of eukaryotic genomes has parallels with ecological communities [3, 4], which vary in the abundance and diversity of species. While it has been shown that niche differences are an important factor in shaping species diversity [5, 6], it is generally believed that neutral processes can explain the assembly of communities over evolutionary time scales [7]. Given the ubiquitous nature of TEs and their contributions to eukaryotic genome evolution [8, 9], an important question is whether or not similar mechanisms operate to shape the genome landscape.

One possible explanation for the variation in TE content and composition between species is that random processes govern the evolution of TE communities and that chance alone determines the outcome for each TE lineage [10]. However, there is strong evidence that TEs integrate in non-random genomic locations, and TEs may show signs of positive selection [11–14]. It is important to understand the phylogenetic distribution of these patterns because TE activity may, in some cases, correlate with the diversification of their host lineages. For example, species radiations in vertebrates appear to be associated with genome repatterning and TE amplification events [15–17]. In one case, the origin of six species of *Taterillus* gerbils within the past 0.4 million years has been accompanied by numerous large chromosomal changes and the non-random accumulation of LINE-1 elements, with the most recently diverged species showing the greatest amount of LINE-1 accumulation [18]. Also, waves of TE amplification are associated with the radiation and subsequent speciation of four genera of salmonid fishes [19]. Similarly, massive retrotransposon amplification appears to coincide with speciation events in hybrid sunflower species [20], and non-random patterns of retrotransposon accumulation in the hybrid species’ genomes indicate a potential mechanism for chromosomal divergence between species [21]. Taken together, these results suggest that studying the properties of TE evolution may indicate the timing and nature of important evolutionary transitions. Thus, we are keenly interested in understanding the nature of TEs in the plant family Asteraceae, which harbors unparalleled species diversity in the plant kingdom [22].

The Asteraceae is the largest family of vascular plants, composed of more than 23,600 species, or 8 % of all plant species [22]. The consensus view is that the Asteraceae originated in South America within the past 40–50 million years, which is somewhat surprising given the large number of species in this family [23]. From South America, the Asteraceae spread to Central America and Africa, and the family currently has a worldwide distribution, being found on every continent except Antarctica [24]. There are 12 recognized subfamilies in the Asteraceae, though four of those subfamilies, the Mutisioideae, Carduoideae, Cichorioideae, and Asteroideae, contain 99 % of the species [24]. Within the Asteraceae, there is exceptional diversity in the ecological distribution of species. For example, there are narrow endemics, and also species such as the common sunflower (*Helianthus annuus*) and dandelion (*Taraxacum officinale*) that are found widely distributed on multiple continents. Though most species in the Asteraceae are herbaceous, there are also many shrub and tree species [24]. However, this plant family is perhaps best known for the numerous agriculturally important species such as cultivated sunflower, safflower, lettuce, and globe artichoke [25]. Given the recent evolutionary origin of this enormous plant family, as well as its global distribution, the Asteraceae represent an excellent system to study plant adaptation and speciation. However, very little is known about genome evolution and TE diversity in the Asteraceae as a whole (but see [26–29]).

In this study, we seek to understand the major features of Asteraceae genomes, and to explore the mechanistic basis of TE evolution in plants by analyzing the evolutionary history of this plant family in a lineage-specific manner. It is known that there is a major bias in genome composition towards *Gypsy* DNA in the common sunflower genome [28, 29], but an outstanding question is whether other Asteraceae genomes exhibit similar patterns. That is, are the genomic properties of the common sunflower unique to that lineage? More importantly, what are the mechanisms contributing to TE community structure in plants? We address these questions by generating whole-genome shotgun (WGS) sequence data from 14 species representing 5 different subfamilies in the Asteraceae, along with an outgroup, and analyzing the relative abundance of TEs in each. We use phylogenetic and linear models to investigate whether there have been lineage-specific patterns of TE evolution in the Asteraceae. We also use ecological measures of community diversity, along with simulation-based approaches, to better understand the genomic impact of TE amplification events and how changes in TE abundance influence TE diversity in the genome as a whole. Taken together, these approaches represent a novel approach to study TE properties by employing descriptive statistical approaches along with phylogenetic and ecological models to investigate the mechanisms of genome community assembly.

## Results

### Transposable element composition in the Asteraceae

**Fig. 1:**
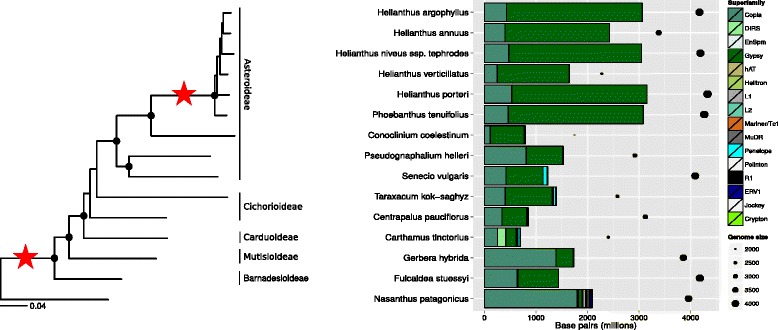
Genomic contribution of TE superfamilies in the Asteraceae. **a** Phylogenetic tree of 14 Asteraceae species and one outgroup species derived from 763 nuclear loci (see Methods). Filled circles indicate nodes with >75 % bootstrap support; to the right of the tree are the subfamilies to which each species belongs; the red stars on the branches indicate the timing of whole genome duplication events based on [56]. **b** Barplot of the genomic composition of TE superfamilies. The x-axis indicates abundance in base pairs for each species, shown along the y-axis. Filled circles indicate the genome size for each species. Superfamilies by order and class: Copia, Gypsy, ERV, and DIRS are LTR-RTs; Helitron is in subclass II of Class II; EnSpm, MuDR, hAT, Mariner/Tc1, and Polinton are TIR Class II TEs; Crypton are unique Class II elements in the order Crypton; L1, L2, and Jockey are LINE non-LTR-RTs; Penelope TEs belong in the unique Penelope order of retrotransposons; R1 are a group of non-LTR-RTs that insert into rDNA genes. The diagonal line through each entry in the barplot legend indicates the border of each TE type in the plot, which is a solid black line

Using WGS sequencing data, we determined that Asteraceae genomes are, on average, composed of 69.9 ± 5.3 % TEs (mean ± SD), with 53.2 ± 19.1 % of these genomes being LTR retrotransposons (LTR-RTs; Fig. 1). As expected for plant species, Class II TEs and non-LTR-RTs were lower in abundance relative to LTR-RTs, comprising just 0.60 ± 0.7 % and 0.82 ± 1.1 % of each genome, respectively. The outgroup species *Nasanthus patagonicus* exhibited comparable patterns of total repeat abundance (62.0 ± 0.1 %) and LTR-RT abundance (47.3 ± 3.3 %) as the Asteraceae, but contained a significantly higher abundance of Class II TEs (2.9 ± 0.1 %; *P =* 0.02) and a higher, though not signicantly so, abundance of non-LTR-RTs (2.0 ± 0.2 %; *P =* 0.20). Interestingly, in all but one species, LINE-like sequences are more prevalent (by a factor of at least 2:1) than other non-LTR-RT types. The one species that does not fit this pattern is *Fulcaldea stuessyi*, a member of the Barnadesioideae (the most basal subfamily of the Asteraceae), which harbors more SINE-like sequences than other non-LTR-RT types. In addition, the *N. patagonicus* genome contains a significantly higher abundance of endogenous retroviruses (ERVs; 1.2 ± 0.4 %; *P =* 0.04) than the average Asteraceae genome (0.06 ± 0.09 %), though it is likely that these sequences represent novel LTR-RTs since plant ERV sequences are more closely related to LTR-RTs than to the *Retroviridae* [30]. Contrasting the widespread nature of the aforementioned TE types, *Penelope* transposons are characterized by a sparse distribution throughout eukaryotes [31]. Consistent with this finding, *Penelope* transposons were found in all but two species in the Asteraceae (*Fulcaldea stuessyi* and *Phoebanthus tenuifolius*), and ERV-like sequences were absent from four species (*F. stuessyi, Conoclinium coelestinum, P. tenuifolius,* and *H. argophyllus*).

**Fig. 2:**
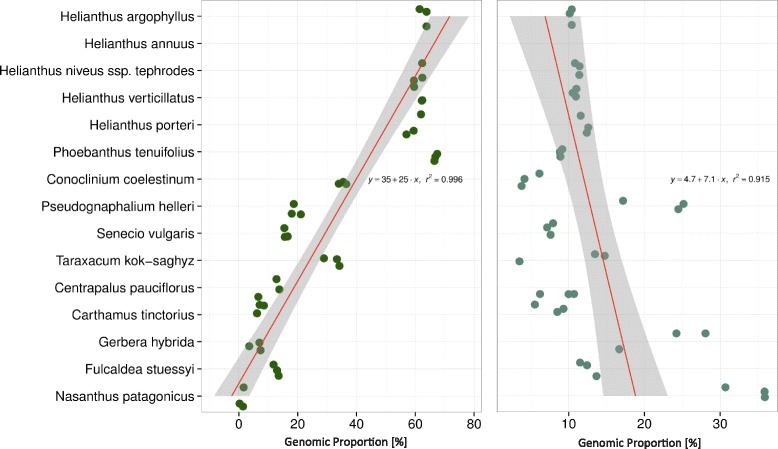
Linear change in genomic composition of LTR-RTs. Shown in phylogenetic order starting with the outgroup (bottom of the y-axis) to the most derived lineages of the Asteraceae in this study (top of the y-axis) are the change in genomic proportion (shown along the x-axis) of *A*) *Gypsy* and *B*) *Copia* TEs

In agreement with previous studies [28, 29], we found a large bias in TE content in the genome of *H. annuus*, which is composed primarily of *Gypsy* elements (60.0 ± 3.3 %)*.* This bias appears to be shared by all members of the subfamily Asteroideae, including all species of the genus *Helianthus* analyzed here (62.4 ± 2.7 %), and the most basal member of the tribe Heliantheae*, P. tenuifolius* (67.5 ± 5.6 %; Fig. 1). We found a significant linear increase in the genomic proportion of *Gypsy* LTR-RTs from the base of the Asteraceae to the most derived subfamily, the Asteroideae using a generalized least squares test (*r*^*2*^ = 0.996; *P* ≤ 2.2e-16; Fig. 2). *Copia* TEs exhibit an inverse pattern to that of *Gypsy*, with species at the base of Asteraceae containing proportionally more *Copia* DNA than those species in the Asteroideae (*r*^*2*^ *=* 0.915; *P* = 2.831e-12; Fig. 2). These phylogenetic patterns remained significant when considering only one *Helianthus* species (*H. annuus*) in the analysis, indicating that they are not due to the overrepresentation of a single genus.

To further investigate the significance of the patterns, we compared the proportion of TEs at the superfamily and family levels along the phylogenetic tree to what would be expected under a Brownian motion model, and we assessed significance of these results using phylogenetically independent contrasts (PICs). We detected significant (*P* < 0.05) phylogenetic signal, *K,* for ten superfamilies of TEs (Additional file 1). Notably, *Copia* TEs as a whole showed significantly (*P <* 0.05) more phylogenetic signal (i.e., *K* ≥ 1) than *Gypsy* (i.e., *K* ≤ 1). At the individual TE family level, we found more LTR-RT families exhibiting significant (*P <* 0.05) phylogenetic signal (7 *Copia* families, 10 *Gypsy* families, 1 *ERV1* family) than either non-LTR-RTs (3 *L1*-like families, 3 *CR1* families, 1, NeSL family) or Class II TEs (1 *hAT* family, 2 *Mariner/Tc1* family, 1 *Helitron* family), though the average phylogenetic signal for Class II TE families was much higher (*K* = 3.26 ± 0) than either LTR-RTs (*K* = 1.78 ± 1.13) or non-LTR-RTs (*K* = 3.19 ± 0.16) [see Additional files 2 and 3].

### Properties of individual TE family evolution

**Fig. 3:**
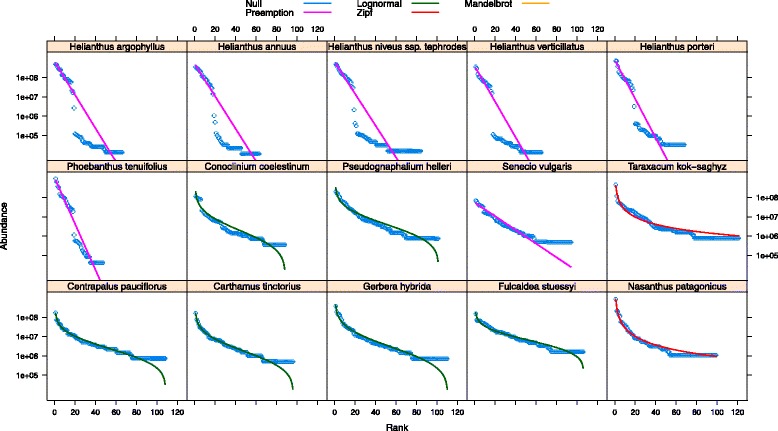
RAD plot of TE family abundance. Species are presented in phylogenetic order starting with the outgroup in the bottom right panel and the moving left to the most derived lineages of the Asteraceae in this study being displayed at the top left. The x-axis depicts the rank order of TEs by abundance, with rank 1 being given to the most abundant family, rank 2 given the second most abundant family, and so on. The y-axis depicts the log abundance of each TE family. Above the plots are the 5 ecological models used to test the fit of observed abundance. The colored line in each panel represents the best-fit model to each distribution as determined by BIC (see Methods)

We investigated the mechanisms of genome community assembly over large time scales by analyzing the rank abundance/dominance (RAD) for all TE families in each species in this study. We considered five ecological models and present the model that best fits the data for each species, as determined by a Bayesian Information Criterion (see Methods). Though numerous species across the Asteraceae exhibit a log-normal-like distribution of TE family abundances (6/15 species), which can be described by even abundances and few rare TE families, it is evident that the predominant pattern is for species to exhibit highly uneven TE family abundances and are thus best fit by a niche-preemption model (7/15 species; Fig. 3). For example, we found that *F. stuessyi*, a member of the subfamily Barnadesioideae, has a very even distribution of TE families in terms of abundance (0.33 ± 0.52 %), while members of the subfamily Asteroideae have a very uneven distribution (see Fig. 1 for subfamily description), being composed of relatively few highly abundant families and many rare families (0.92 ± 2.4 %). Six species in the Heliantheae show TE family distributions best fit by a straight line (i.e., the niche preemption model; Fig. 3). The dominance of TE families in the Heliantheae is evident when considering that the top 10 TE families in this group account for nearly 2X the genomic proportion (51.5 ± 3.14 %) as the top 10 TE families in the rest of the Asteraceae (26.8 ± 9.10 %).

**Fig. 4:**
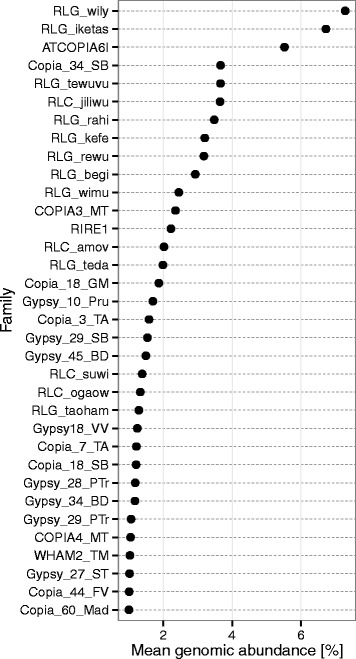
Rank abundance of TE families in the Asteraceae. The y-axis depicts the most abundant TE families in the Asteraceae, listed in decreasing rank abundance from the top the y-axis. The x-axis shows the average percent genomic abundance of each TE family in the Asteraceae

**Fig. 5:**
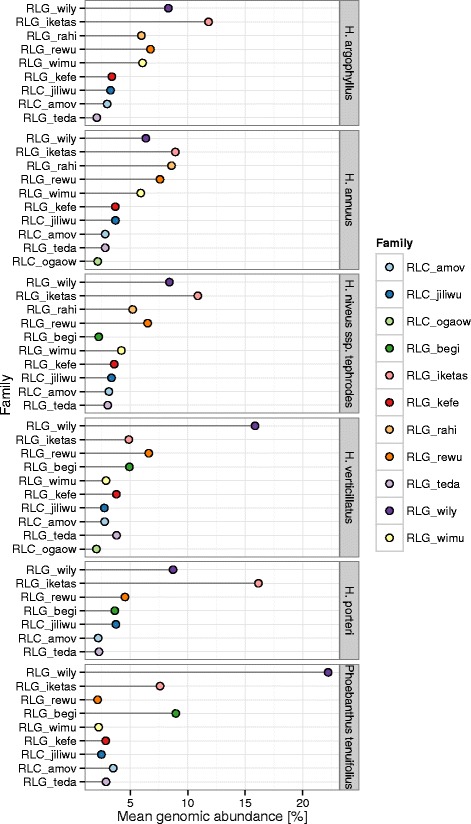
Rank abundance of TE families in the Heliantheae. Along the y-axis is the rank abundance of the top 2 % of TE families in the Heliantheae, in decreasing order. Each panel depicts the rank abundance of TE families in phylogenetic order of the tribe from the base of the plot. The x-axis shows the percent genomic abundance of each TE family

While the RAD models described above demonstrate global patterns of abundance and dominance of TE families, these plots are unlabeled and do not allow investigation of specific changes in rank abundance. To infer which specific TE families have contributed the most to the rank abundance patterns observed in this study, and in the marked change in rank abundance and dominance within the Heliantheae in particular, we analyzed the rank of TE families sorted by abundance in the Asteraceae as a whole (Fig. 4) as compared to the abundance of TE families within the Heliantheae (Fig. 5). Interestingly, we found no phylogenetic patterns of rank abundance at the TE family level that are shared across the Heliantheae (Fig. 5). At the superfamily level, however, it is clear that at least the four highest-ranking TE families in the each species in the Heliantheae are members of the *Gypsy* superfamily.

### Impact of TE family abundance on TE diversity

**Fig. 6:**
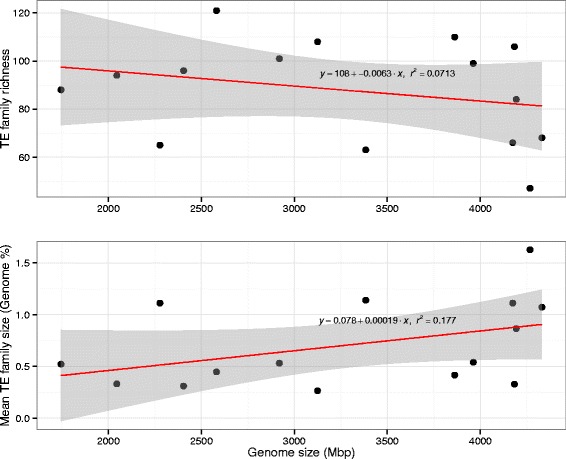
Relationship between genome size and TE family size and richness. Along the x-axis is shown the genome size of each species in mega-base pairs. **a** The TE richness, or total number of TE families seen, is shown along the y-axis. **b** The mean TE family size as a percent of the genome is depicted on the y-axis

**Fig. 7:**
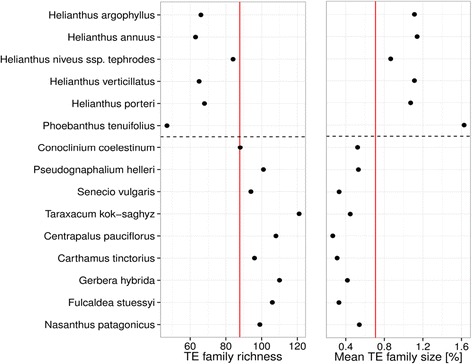
Phylogenetic relationship between TE richness and TE family size. **a** The TE family richness is shown along the x-axis for each species, which are depicted in phylogenetic order from the outgroup species at the base of the y-axis to the most derived lineages in the Asteraceae at the top of the y-axis. **b** The mean TE family size as a percentage of the genome is shown along the x-axis. In both panels, the red vertical line indicates the mean and the horizontal dashed black line shows the base of the Heliantheae (with all species in the Heliantheae being shown above the line)

To investigate the potential impact of changes in TE abundance on patterns of genome community diversity, we estimated the correlation of changes in TE family abundance and TE richness with genome size. As expected for plant species [1, 32, 33], the abundance of retrotransposon DNA is strongly correlated with genome size (*r*^*2*^ *=* 0.608; *P* = 6.06e-4; Additional file 4). These patterns were also significant when considering the non-independence of the species with a phylogenetic generalized least squares test (*Copia*, *P* = 0.0009; *Gypsy*, *P* = <0.0001; Additional file 5). However, while we did find a positive correlation with genome size and TE family size, we did not find such a correlation with genome size and TE richness (Fig. 6). To investigate the impact of genome dominance by some TE families on genome community structure, we also calculated Shannon’s diversity and evenness of TE families for each species in this study (Additional file 6), which may provide more insight into the evolution of genome community patterns than looking at TE richness alone [34]. For example, in addition to the major shift in genome composition at the base of Heliantheae, there also appears to be a reduction in Shannon’s diversity and evenness (Additional file 6). This result is further supported by a marked increase in the average TE family size in the Heliantheae, which is accompanied by a decrease in TE richness (Fig. 7).

## Discussion

It is well known that TEs vary in abundance and type between eukaryotic species. For example, TEs are completely absent from the genomes of some unicellular eukaryotes [36], though >50 % of the human genome is composed of TEs [35]. Similarly, the TE composition of the *Saccharomyces cerevisiae* genome is 4 % [37], and includes only LTR-RTs, whereas some plant genomes are >80 % TEs e.g., [29, 38–40], including hundreds of families of both Class I and Class II TEs [12]. There is also a disparity with respect to TE copy number and the occurrence of contemporary TE activity. For example, mammalian genomes contain numerous high copy number TE families though only a few recently active TE families have been discovered [41]. Conversely, there are many active TE families in the genomes of fruitfiles and pufferfish, but these families only contain a few copies [42–44]. Given the potential impact of TEs on genome structure and gene expression divergence [45–47] and the apparent variation in TE susceptibility amongst eukaryotes, an understanding of the timescales and phylogenetic patterns over which different classes of TEs are active is of great interest.

### Transposable elements and genome content in the Asteraceae

Species in the Asteraceae vary tremendously in the TE composition of their genomes, especially with respect to LTR-RTs (Fig. 1). It is not surprising that the greatest magnitude of change in genome content involves LTR-RTs given that these sequences account for the largest portion of each genome. It is, however, interesting that we see such strong linear patterns of change in genome content at the LTR-RT superfamily level from the base of the Asteraceae to the crown lineages (Fig. 2). In the broad sense, these patterns fit the expectation of zero-sum change for a neutral community, which predicts that an increase in abundance in one member of a community will result in a proportional decrease in the abundance of another [7]. Though TE activity may lead to expansion of the nuclear genome [20, 38, 48], the inverse patterns of change in *Gypsy* and *Copia* abundance in the Asteraceae reflects that there are a finite number of insertion sites in the genome, and increases in copy number of one or more TE families may result in the replacement or inactivation of other TE copies.

We detected significant phylogenetic signal for both Class I and Class II TEs at both the superfamily and family level (Additional files 1, 2 and 3), indicating that the genomes of related species are more similar in TE composition and abundance than expected by chance. When considering the variation in genome content between the basal and most derived lineages of the Asteraceae (Fig. 1), this result is expected. However, it seems likely that very different processes contributed to these phylogenetic patterns. For example, the phylogenetic signal seen in *Penelope* retrotransposons and ERVs may be a product of the sparse distribution of those sequences. The genomic composition of ERVs in *N. patagonicus* appears high relative to the Asteraceae, though this finding not uncommon for plant species. For example, the genomic percentage of ERVs is 2.4 % in the *Amborella* genome [49], twice that of *N. patagonicus.* Alternatively, *Gypsy* elements are found in all species in the Asteraceae, but there is a clear increase in the abundance of several *Gypsy* families at the base of Heliantheae, producing a phylogenetic pattern shared by all members of this tribe. The inverse pattern can be seen for the *Copia* superfamily, which also shows significant phylogenetic signal (Additional file 1), where a linear decrease in these sequences from the Barnadesioideae to the Asteroideae contributes to phylogenetic patterns across the family. The foregoing results indicate that no single evolutionary process can explain these patterns of genome evolution in the Asteraceae. Specifically, species in the basal subfamilies of the Asteraceae are strikingly different in TE composition compared with the crown subfamilies, with those species in the basal subfamilies containing a greater abundance of non-LTR-RTs and DNA transposons. Could the greater TE diversity at the base of the Asteraceae and in the outgroup species be a result of the age of those lineages, or could there be other mechanisms influencing the abundance and diversity of the genome community? While it is not currently possible for us describe the evolutionary events that produced these patterns, ongoing genome sequencing projects in the Asteraceae should enable better descriptions in future studies.

### Transposable element families and genome community assembly

Although ecosystems typically vary in terms of their species abundance and diversity, most communities exhibit a very similar distribution in the relative abundance of species [7]. Specifically, most communities exhibit a log-normal-like distribution of species abundance, with few species having high abundance, many rare species with very low abundance, and numerous species lying between these extremes [7]. Interestingly, one prior study has shown that eukaryotic genomes appear to exhibit similar log-normal distributions of genetic elements, suggesting that neutral processes may best explain community assembly over evolutionary timescales, regardless of the system [50]. However, there is some doubt as to whether the log normal model is the best null hypothesis for TE abundance distributions [51]. We tested a range of neutral and niche-based abundance distribution models and asked whether Asteraceae genomes also exhibit a log-normal distribution of TE family abundances, and whether there are shared patterns of TE abundance distributions across the family. While six species in this study exhibit a log normal distribution of TE abundance, a greater number, seven species, exhibit a niche-preemption distrbution, and two species have a TE abundance distribution best fit by the Zipf model, a hierarchical distribution (Fig. 3).

Interestingly, there is a very marked break at the base of Heliantheae with all species in this tribe exhibiting numerous highly abundant TE families and many rare families. This type of distribution has been used to describe communities with poor habitat [52] and/or few species [53], or the early succession of species [54] following disturbance [55]. Typically, these patterns of uneven abundance do not fit neutral expectations [56]. While there are caveats in interpreting ecological models in a genomic context, these results, taken together with other measures of TE abundance presented here, clearly reflect a unique evolutionary history for this tribe.

What biological change facilitated the major genomic transitions in the Heliantheae? It is tempting to speculate that the whole genome duplication event at the base of the Heliantheae [57] may have provided a genomic disturbance which contributed to the biased distribution of TE family abundance in this tribe, or directed integration of *Gypsy* elements may have contributed to these patterns [11, 29, 58]. Clearly, more work will be required to gain a deeper understanding of the underlying processes. It is clear from this analysis, however, that whole-genome turnover and expansion events have taken place in the lineage leading to the tribe Heliantheae, which arose ca. 26–31 MYA [57, 59].

### Mechanisms of change in the genome-wide level of transposable elements

Major transitions in genome content are evident in each subfamily of the Asteraceae (Fig. 1). What is the best mechanistic explanation of the patterns of TE abundance in the Asteraceae? The coexistence of species may be facilitated by niche differentiation [60], and this type of model best explains the TE abundance data we see for species in the tribe Heliantheae. However, the TE abundance and diversity for this group of species indicates a very biased composition towards *Gypsy* TEs (Figs. 2 and 3). The linear increase in abundance of *Gypsy* TEs in the Asteraceae has had at least two major influences on the genome community of TEs. First, the correlation we see with TE family size and genome size (Fig. 6) indicates an unequal contribution of TE families to the genome community. Second, it is clear that the linear pattern of increase in *Gypsy* is driven by only a few TE families (Fig. 3), which has lead to an increase in average family size and a decrease in overall TE richness (Fig. 7). Interestingly, we do not see different superfamilies dominating *Helianthus* genomes as has been observed in some species of *Gossypium* [61]. This may indicate that a single event at the base of Heliantheae produced the observed genomic change, and that the patterns we see in each *Helianthus* species are shared by phylogenetic history rather than being independent events leading to similar patterns in each species. Alternatively, *Gypsy*elements may have evolved features allowing them to outcompete other TEs or avoid host-silencing mechanisms. Future investigations into these questions will surely lead to a greater understanding of the processes contributing to the high levels of diversity observed within the Asteraceae, and to the processes contributing to the evolution of TE diversity across the plant kingdom as a whole.

## Conclusions

The majority view of TE evolution is that these sequences evolve primarily by neutral processes and are therefore likely to generate predictable distributions of relative abundance [50]. We showed, however, that plant species may exhibit uneven distributions of TE family abundance, as exemplified by all members of the Heliantheae investigated herein. Our results indicate that these patterns may be facilitated by: 1) an unequal contribution of certain TE families over time [29, 62]; and 2) nonrandom patterns of TE accumulation across the genome, as has been shown for one species in this study, *H. annuus* [21, 26, 27]. Aside from species in the tribe Heliantheae, other species in the Asteraceae do exhibit TE abundance distributions that are in line with neutral expectations. This finding may indicate that the factors contributing to the relative abundance TEs vary over time. Based on these results, we believe that the relative abundance of TEs in plant genomes can be best described as a continuum of resource-based patterns (i.e., niche-preemption) to random patterns (i.e., neutral processes). Our finding of major shifts in TE composition at the base of the Asteraceae and at the base of the tribe Heliantheae provides further evidence that TE compositions contain phylogenetic signal [63], and suggests a possible role for TEs in species formation in the Asteraceae.

## Methods

### Taxon sampling and WGS sequencing

To investigate patterns of genome evolution across the Asteraceae, we generated paired-end Illumina Hi-Seq sequence data (100 bp in length; 400 bp insert size) for individuals from 15 taxa. The estimated genome coverage for each species ranged from 0.42x – 3.52x (Additional file 7). These species were selected to represent every major subfamily of the Asteraceae, and included an outgroup species, *N. patagonicus* (Additional file 7). In addition, five of the taxa were selected from the genus *Helianthus* in order to investigate patterns of genome evolution amongst closely related species, and to increase our understanding of the evolutionary history of the most well-studied species in the family, *H. annuus*, for which there have been numerous prior studies about TE properties (see [26–29]). This study was done in parallel with a previously published phylogenomic study in which the taxon sampling and library preparation methods are described [64].

### Repeat identification from WGS sequences

Prior to analysis, all WGS reads were treated with PRINSEQ version 0.19.4; [65] with the parameters ‘-min_len 40 –noniupac –min_qual_mean 15 –lc_method entropy –lc_threshold 60 –trim_ns_right 10 –ns_max_p 20’ to remove low quality and short sequences. After quality filtering, we screened all chloroplast- and mitochondria-derived sequences from the WGS reads using the complete chloroplast genome sequence for cultivated sunflower line HA383 (Genbank accession number DQ383815) and a database of 10 complete plant mitochondria genome sequences obtained from Genbank, respectively. One million paired-end reads were sampled randomly from each set of screened reads and interleaved with Pairfq version 0.09; [66] prior to analysis. Repeat identification was carried out by performing an all-by-all BLAST following the methods of Staton et al. [29] with the 1 million randomly sampled paired-end reads, followed by clustering using the Louvain method [67]. Annotation of clusters was performed using blastn [68] against RepBase version 18.01; [69] and a set of full-length LTR-RTs described by Staton et al. [29]. Our repeat identification methods are implemented using the Transposome software version 0.03; [70] that we developed for this study. We performed three replicates of the above sampling and annotation procedure with Transposome for each species to minimize the statistical error in our estimates of genome composition.

To investigate the effect of varying levels of genome coverage, we simulated 10 different levels of genome coverage from the *H. annuus* WGS reads ranging from 0.056 to 5.1 %, with 3 replicates at each level (total of 30 read sets). The coefficient of variation in the inferred genomic composition of each TE family was measured at each level of genome coverage after analysis with Transposome to infer the appropriate level of sampling; this allowed us to maximize the level of TE diversity being captured.

### Genome size estimation and prediction of changes in genome composition

In order to determine the genomic contribution of each TE family to the species in this study, and estimate the magnitude of change across the Asteraceae, we calculated genome size according to Hu et al. [71], with modifications. Using WU-BLAST with parameters “M = 1 N = -3 -Q -R 1” we mapped a reference transcriptome of 11 species from the Compositae Genome Project database (http://compgenomics.ucdavis.edu/) to 5 million WGS reads for each species, and calculated the coverage of each transcript using the formula:

$$ Co{v}_i=N/L $$

where *N* is the total length of reads mapped and *L* is the transcript length. The genome size (*Cval*) for each species was then determined by the formula:

$$ Cval=P\times \left(n\times l/ mean\left(Co{v}_i\right)\right) $$

where *P* is the ploidy level, *n* is the total number of reads, and *l* is the read length. In the above formula, only alignments over 60 base pairs in length and over 70 % identity were considered. These values were chosen from a permutation test using all possible alignments from lengths 50–100 and percent identity thresholds from 50 to 100, comparing observed to expected values. The mean coverage (*Covi*) was trimmed to remove the top 10 % of transcripts by coverage. The estimated genome size for each species, along with the published prediction (if available), is shown in Additional files 7 and 8.

The genomic contribution of each TE superfamily was calculated from the annotation summary file generated by Transposome (Fig. 1), and was used to determine the magnitude of change in TE composition in each species. Generalized least squares tests were performed with the R programming language [72] to estimate directional change in TE content in the Asteraceae (Fig. 2). We calculated Shannon’s evenness and diversity statistics using the R package Vegan [73] to investigate the influence of genome size change on TE diversity statistics.

### Phylogenetic patterns of TE family evolution

In addition to analyzing statistical patterns of repeat abundance, we also explored a mechanistic basis for TE evolution in the Asteraceae from an ecological perspective through the use of community ecology models. First, we compared RAD distributions using the R package Vegan [73] to investigate the processes leading to the inferred distribution of TE families in the Asteraceae [50]. We compared five ecological models to test whether the rank abundance distribution of TE families in each species was best fit by neutral or niche-based models (reviewed in [56]). As in previous studies (e.g., [4, 74]), we treat a TE family as analgous to a biological species, the genome as analagous to the ecological communtiy, and an individual TE is treated as an individual of a given species. The Null model fits a brokenstick model where individual TEs are randomly distributed among the observed TE families and no parameters are fitted [5]. The Lognormal and Zipf models are generalized linear models where the Lognormal model assumes the logarithm of abundances are distributed normally [73]. The Zipf model,

$$ {a}_r=Jp{r}^Y, $$

where *a* is the expected abunance of a TE family at rank *r*, *J* is the total number of individual TEs, *p* is the fitted proportion of the most abundant TE family, and *Υ* is a decay coefficient, is used to fit a particular power law distribution [73]. The Mandelbrot model is a generalization of the Zipf model and adds one nonlinear parameter to the Zipf with the remaining parameters and log-likelihood being fitted with a linear model [73]. In the Preemption model, also called the geometric series or niche preemption model, each level of TE family abundance is a sequential, constant proportion of the total number of individuals in the whole community. The preemption model rank abundance is fit by straight line in the RAD plot [75].

Second, a phylogenetic generalized least squares (pgls) test was conducted using caper [76] to test for the association of changes in TE composition with particular phylogenetic divisions within the Asteraceae and genome size. The phylogenetic tree used in the pgls analyses was generated from an alignment of 763 nuclear loci sequenced by a novel targeted enrichment method [64]. The model we tested was:

$$ Log\left( Genome\  size\right)\sim Log\left(S*\right) $$

where S* is the superfamily percent genomic abundance.

To further investigate the mechanisms and timing of shifts in genome content, we calculated phylogenetic signal for each TE family by using a descriptive statistic called *K,* which indicates significant phylogenetic signal for a trait, in this case TE abundance, on the tree compared to a Brownian motion model, along with phylogenetic independent contrasts PICs; [77, 78]. These calculations were performed using the R package picante [79], and all statistical analyses and plotting were performed in R [72].

### Data availability

All sequence data in this paper is deposited in the NCBI Short Read Archive under BioProject number PRJNA288472.

## Additional files

Additional file 1:
**Displays the phylogenetic signal for TE superfamilies in the Asteraceae.** (PDF 11 kb) Additional file 2:
**Displays the phylogenetic signal for TE superfamilies.** (PDF 61 kb) Additional file 3:
**Shows the TE families exhibiting significant phylogenetic signal.** (PDF 63 kb) Additional file 4:
**Depicts the relationship between retrotransposon DNA and genome size.** (PDF 63 kb) Additional file 5:
**Shows results from GLS and PGLS tests for the evolution of** ***Gypsy*** **and** ***Copia*** **composition.** (PDF 60 kb) Additional file 6:
**Shows the genome diversity statistics for TE families.** (PDF 807 kb) Additional file 7:
**Shows the raw data statistics and genome size estimates.** (PDF 66 kb) Additional file 8:
**Shows published genome size estimates and genome size observations determined by the method described in this study.** (PDF 54 kb)

## Abbreviations

TE: Transposable element
LTR: Long terminal repeat
LTR-RT: Long terminal repeat retrotransposon
ERV: Endogenous retrovirus
LINE: Long interpersed nuclear element
SINE: Short interspersed nuclear element
WGS: Whole-genome shotgun, SD, Standard deviation
PIC: Phylogenetic independent contrast
RAD: Rank abundance dominance
MYA: Million years ago
