# Supplementary figures and images for: Evolutionary transitions in the Asteraceae coincide with marked shifts in transposable element abundance
Source: BMC Genomics. 2015 Aug 20;16(1):623. doi: 10.1186/s12864-015-1830-8 (PMC4546089; doi:10.1186/s12864-015-1830-8)

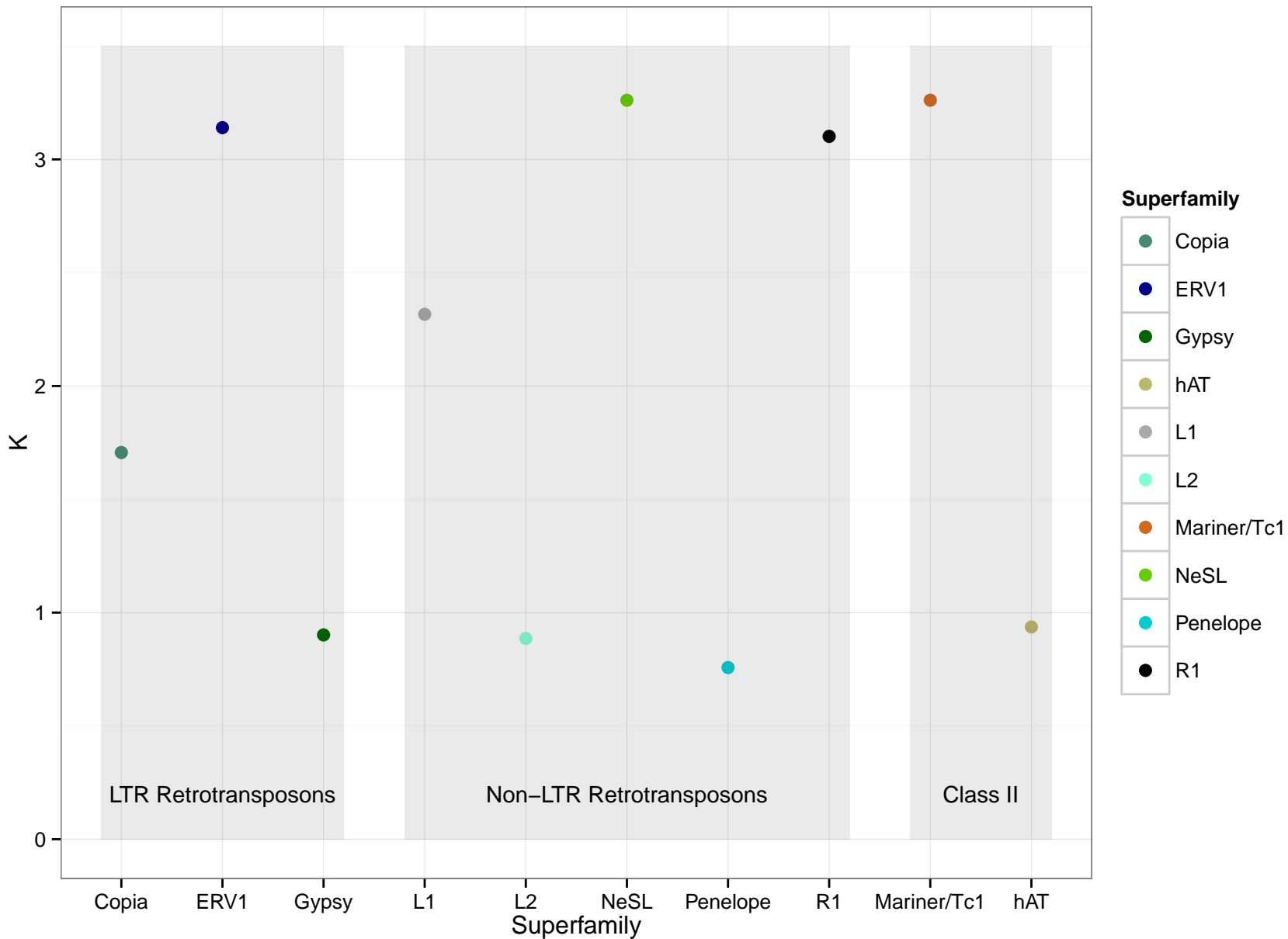

Supplement: Additional file 1: — Displays the phylogenetic signal for TE superfamilies in the Asteraceae. (PDF 11 kb) [file 12864_2015_1830_MOESM1_ESM.pdf]
