# Supplementary material for: Evolutionary transitions in the Asteraceae coincide with marked shifts in transposable element abundance
Source: BMC Genomics. 2015 Aug 20;16(1):623. doi: 10.1186/s12864-015-1830-8 (PMC4546089; doi:10.1186/s12864-015-1830-8)
Supplement: Additional file 2: — Displays the phylogenetic signal for TE superfamilies. (PDF 61 kb) [file 12864_2015_1830_MOESM2_ESM.pdf]

Additional file 2. Phylogenetic signal for TE families in the Asteraceae. Shown are TE families exhibiting significant phylogenetic signal as compared to a Brownian motion model of evolution along the phylogenetic tree. For each TE family, we demonstrate the observed PIC (phylogenetic independent contrast) scores and significance value, along with the value of the random PIC scores.

Order	Superfamily	Family	K	PIC variance (observed)	PIC variance (random mean)	PIC variance *P-value*	PIC variance *Z-value*
LTR-RT	Copia	COPIA2_LC	3.257875889	5.14E+15	3.67E+16	0.039	-0.909125888
LTR-RT	Copia	COPIA2_MT	3.30773527	1.17E+16	8.03E+16	0.008	-0.878074852
LTR-RT	Copia	Copia15_VV	1.302072147	7.08E+13	3.73E+14	0.018	-2.10973762
LTR-RT	Copia	RLC_X	2.663159151	2.05E+17	1.26E+18	0.019	-0.89629912
LTR-RT	Copia	RLC_amov	0.710946644	2.98E+16	7.74E+16	0.037	-1.907217855
LTR-RT	Copia	RLC_jiliwu	0.520839198	1.07E+17	3.06E+17	0.018	-1.210112268
LTR-RT	Copia	RLC_ogaow	0.938232015	7.84E+15	2.94E+16	0.022	-2.438721569
LTR-RT	Gypsy	RLG_X	0.703019737	2.05E+17	1.26E+18	0.019	-0.89629912
LTR-RT	Gypsy	RLG_kefe	1.076403493	2.52E+16	9.59E+16	0.002	-2.767755405
LTR-RT	Gypsy	RLG_rewu	0.726242862	1.10E+17	3.30E+17	0.049	-2.032258579
LTR-RT	Gypsy	RLG_ryse	0.661494372	1.95E+15	4.56E+15	0.025	-1.933088881
LTR-RT	Gypsy	RLG_teda	1.507538406	1.25E+16	7.55E+16	0.006	-3.239986584
LTR-RT	Gypsy	RLG_tewuvu	0.850900791	7.53E+16	3.50E+17	0.011	-0.931314665
LTR-RT	Gypsy	DM176	3.261186883	3.34E+11	2.18E+12	0.039	-0.836834688
LTR-RT	Gypsy	GYPSY16_AG	3.261186883	3.34E+11	2.18E+12	0.036	-0.848736222
LTR-RT	Gypsy	Gypsy123_DR	3.239593367	1.34E+12	9.04E+12	0.0495	-0.881410277
LTR-RT	Gypsy	Gypsy1_SM	0.870785834	9.46E+11	5.12E+12	0.043	-1.03495498
LTR-RT	ERV1	ERV1_N6_DR	3.210894013	3.43E+14	2.29E+15	0.0335	-0.875115482
Non-LTR-RT	L1	L1_11_DR	2.79895668	2.60E+13	1.63E+14	0.01	-1.042094132
Non-LTR-RT	L1	L1_12_DR	3.261186883	3.34E+11	2.41E+12	0.0355	-0.928489469
Non-LTR-RT	L1	L1_58_ACar	3.261186883	5.35E+12	3.61E+13	0.0395	-0.866658002
Non-LTR-RT	NeSL	LIN4b_SM	3.261186883	3.34E+11	2.15E+12	0.0365	-0.832815617
Non-LTR-RT	CR1	CR1_13_CQ	3.261186883	3.34E+11	2.23E+1	0.0385	-0.868749537
Non-LTR-RT	CR1	CR1_58_HM	3.261186883	3.34E+11	2.23E+12	0.036	-0.872823223
Non-LTR-RT	CR1	CR1_79_HM	3.261186883	2.14E+13	1.46E+14	0.0325	-0.870677374
Class II	hAT	P4_AG	3.261186883	3.34E+11	2.30E+12	0.0255	-0.892685921
Class II	Mariner/Tc1	SMAR15	3.261186883	3.01E+12	1.99E+13	0.0375	-0.854157409
Class II	Mariner/Tc1	ATHPOGON1	3.261186883	1.21E+14	7.69E+14	0.0355	-0.830930169
Class II	Helitron	Helitron3_PPa	3.261186883	5.35E+12	3.45E+13	0.0405	-0.841984529
