# Supplementary material for: Evolutionary transitions in the Asteraceae coincide with marked shifts in transposable element abundance
Source: BMC Genomics. 2015 Aug 20;16(1):623. doi: 10.1186/s12864-015-1830-8 (PMC4546089; doi:10.1186/s12864-015-1830-8)
Supplement: Additional file 3: — Shows the TE families exhibiting significant phylogenetic signal. (PDF 63 kb) [file 12864_2015_1830_MOESM3_ESM.pdf]

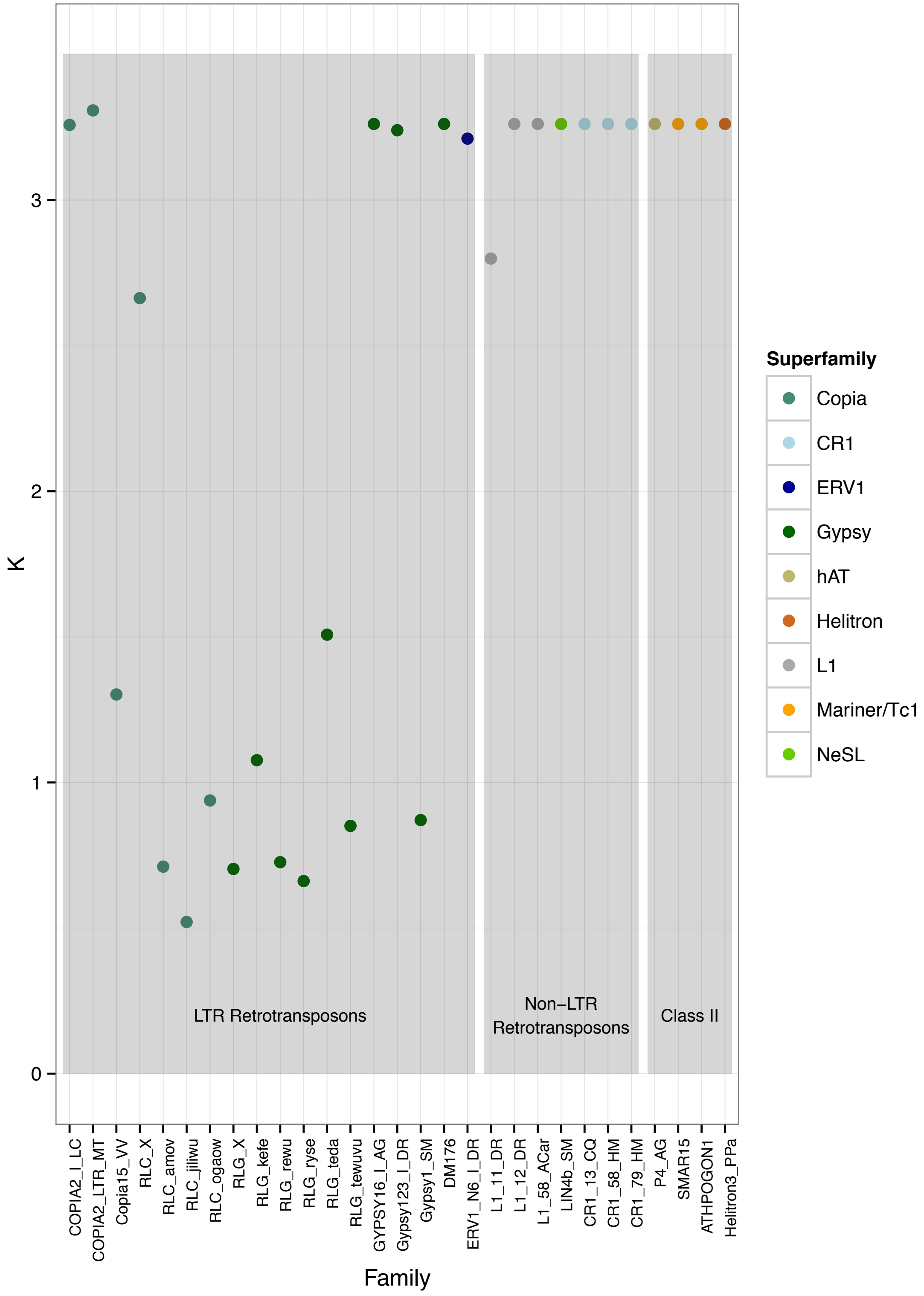

Additional file 3. TE families exhibiting significant phylogenetic signal. Along the x-axis are TE families in alphabetical order (divided by order, which is indicated by gray boxes) exhibiting significant phyologenetic signal (y-axis).
