# Supplementary material for: Evolutionary transitions in the Asteraceae coincide with marked shifts in transposable element abundance
Source: BMC Genomics. 2015 Aug 20;16(1):623. doi: 10.1186/s12864-015-1830-8 (PMC4546089; doi:10.1186/s12864-015-1830-8)
Supplement: Additional file 4: — Depicts the relationship between retrotransposon DNA and genome size. (PDF 63 kb) [file 12864_2015_1830_MOESM4_ESM.pdf]

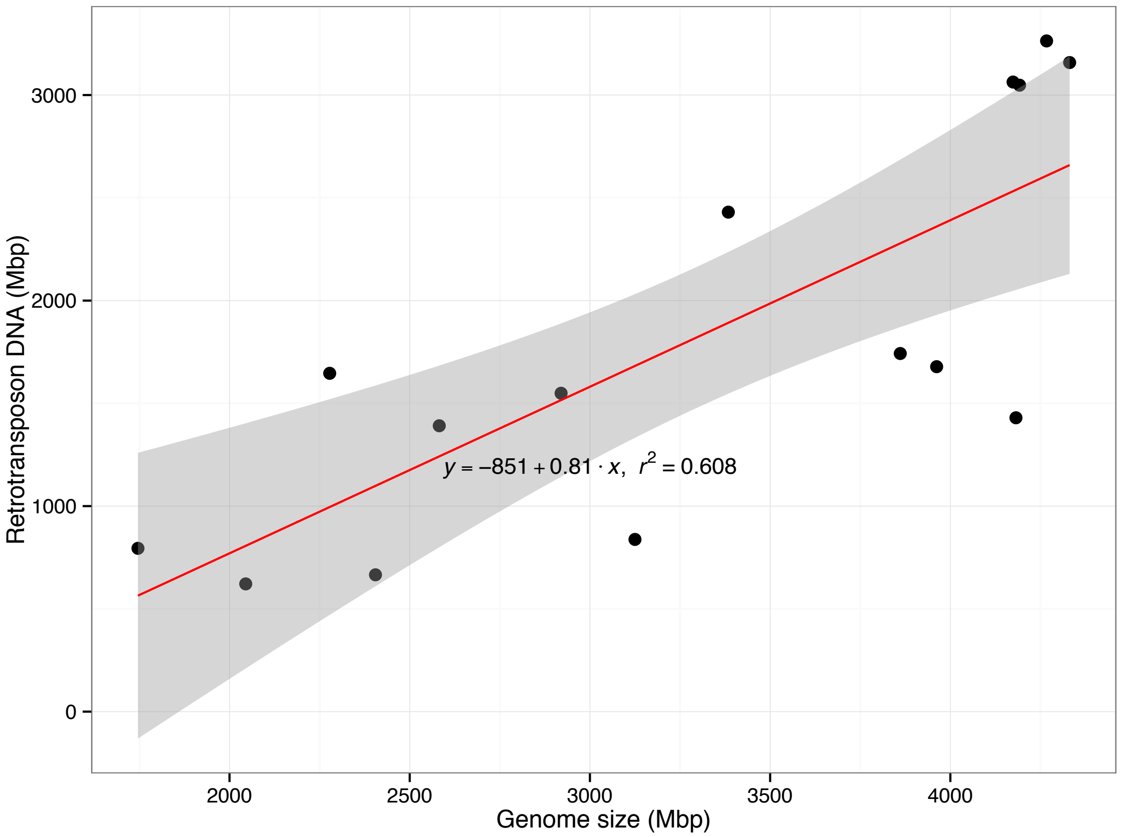

Additional file 4. Relationship between retrotransposon DNA and genome size. The total amount of retrotransposon base pairs (y-axis) correlates very strongly with genome size (y-axis) in the Asteraceae.
