# Supplementary material for: Evolutionary transitions in the Asteraceae coincide with marked shifts in transposable element abundance
Source: BMC Genomics. 2015 Aug 20;16(1):623. doi: 10.1186/s12864-015-1830-8 (PMC4546089; doi:10.1186/s12864-015-1830-8)
Supplement: Additional file 5: — Shows results from GLS and PGLS tests for the evolution of Gypsy and Copia composition. (PDF 60 kb) [file 12864_2015_1830_MOESM5_ESM.pdf]

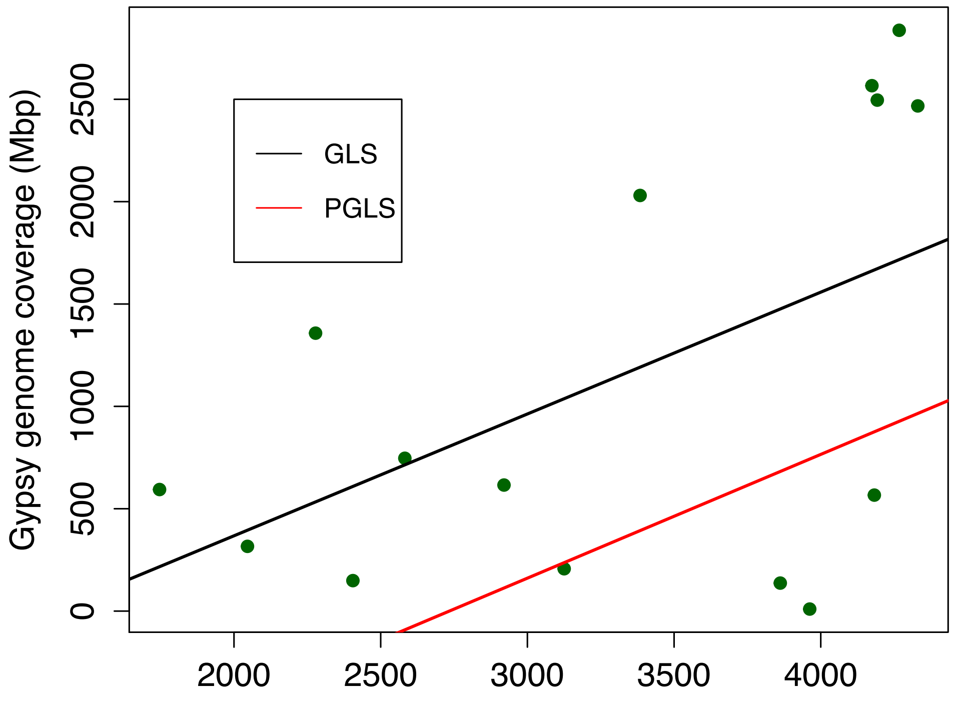

Gypsy genome coverage (Mbp)
0
500
1000
1500
2000
2500
2000
2500
3000
3500
4000
GLS
PGLS

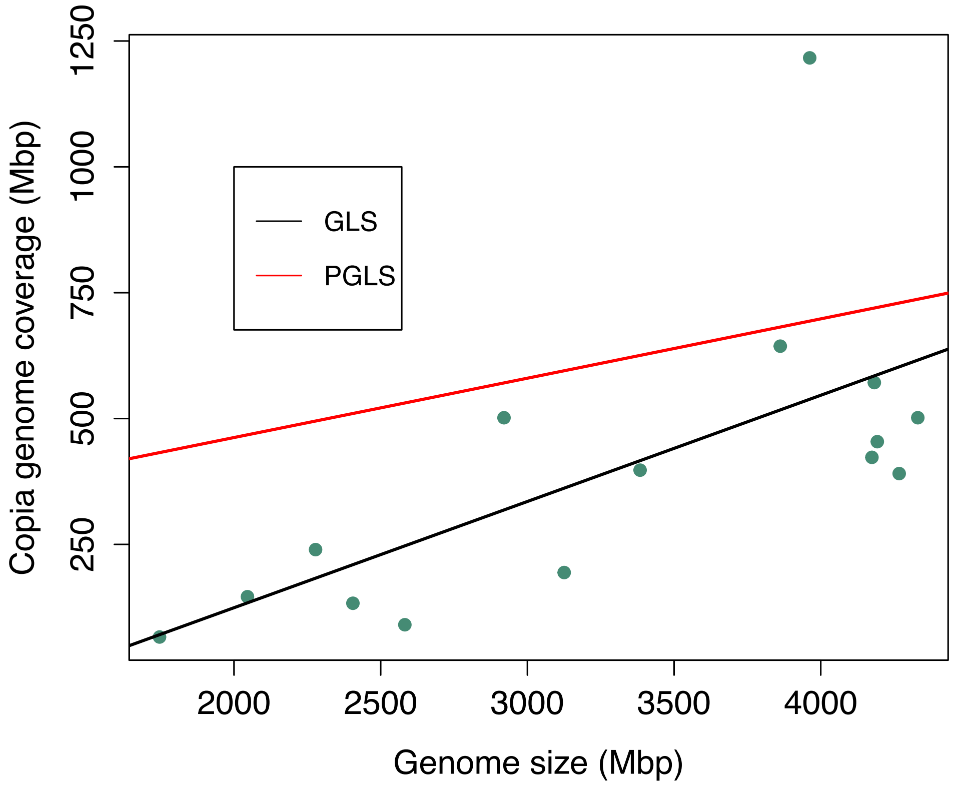

Copia genome coverage (Mbp)
250
500
750
1000
1250
2000
2500
3000
3500
4000
Genome size (Mbp)
GLS
PGLS

Additional file 5. GLS and PGLS tests for the evolution of *Gypsy* and *Copia* composition. The genomic composition (y-axis) of A) *Gypsy* and B) *Copia* TEs correlates strongly with genome size (x-axis) as shown by the GLS fit (black line), even when considering the phylogenetic relatedness of the species with a PGLS test (red line).
