# Supplementary material for: Evolutionary transitions in the Asteraceae coincide with marked shifts in transposable element abundance
Source: BMC Genomics. 2015 Aug 20;16(1):623. doi: 10.1186/s12864-015-1830-8 (PMC4546089; doi:10.1186/s12864-015-1830-8)
Supplement: Additional file 6: — Shows the genome diversity statistics for TE families. (PDF 807 kb) [file 12864_2015_1830_MOESM6_ESM.pdf]

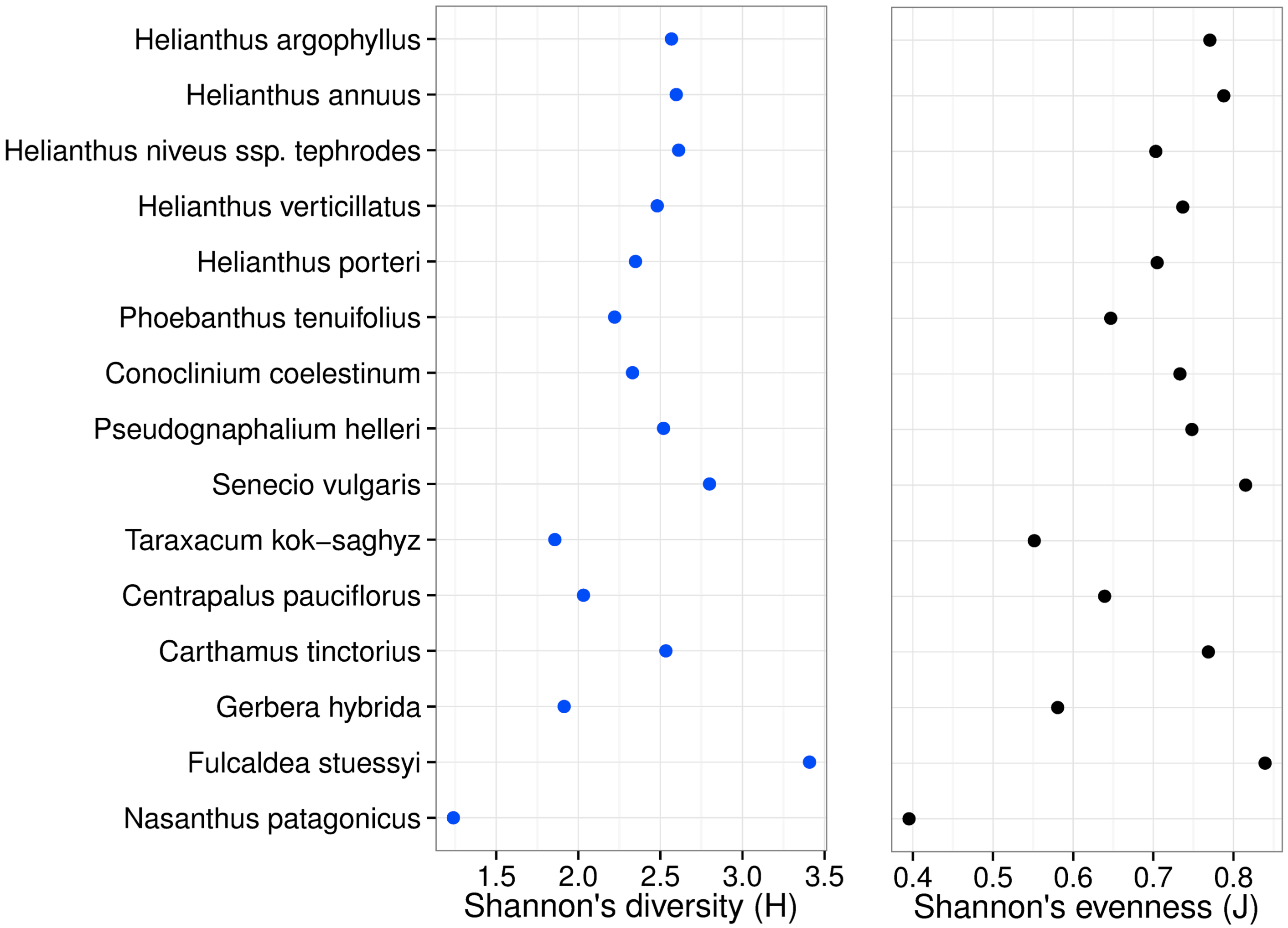
Helianthus argophyllus
Helianthus annuus
Helianthus niveus ssp. tephrodes
Helianthus verticillatus
Helianthus porteri
Phoebanthus tenuifolius
Conoclinium coelestinum
Pseudognaphalium helleri
Senecio vulgaris
Taraxacum kok–saghyz
Centrapalus pauciflorus
Carthamus tinctorius
Gerbera hybrida
Fulcaldea stuessyi
Nasanthus patagonicus
1.5
2.0
2.5
3.0
3.5
Shannon's diversity (H)
0.4
0.5
0.6
0.7
0.8
Shannon's evenness (J)

Additional file 6. Genome diversity statistics for TE families. The species shown along on the y-axis are in phylogenetic order from the outgroup (base of the y-axis) to the most derived lineages of the Asteraceae (top of the y-axis). The filled blue points are Shannon's diversity (H), and the black points show Shannon's evenness (J).
