# Supplementary material for: Evolutionary transitions in the Asteraceae coincide with marked shifts in transposable element abundance
Source: BMC Genomics. 2015 Aug 20;16(1):623. doi: 10.1186/s12864-015-1830-8 (PMC4546089; doi:10.1186/s12864-015-1830-8)
Supplement: Additional file 7: — Shows the raw data statistics and genome size estimates. (PDF 66 kb) [file 12864_2015_1830_MOESM7_ESM.pdf]

Additional file 7. Raw data statistics and genome size estimates. For each species in this study, we show the number of sequence reads generated and the corresponding genome coverage obtained from the genome size estimates (see Methods).

Subfamily	Tribe	Genus	Species	Genome size [1]	Num. reads/ Genome X-coverage
Calyceraceae	Calyceraceae	*Nastanthus*	*patagonicus*	3962340892	22733114/0.58
Barnadesioideae	Barnadesieae	*Fulcaldea*	*stuessyi*	4182557218	92343086/2.23
Mutisioideae	Mutisieae	*Gerbera*	*hybrida*	3861919879	19128428/0.50
Carduoideae	Cardueae	*Carthamus*	*tinctorius*	2405291468	18020913/0.76
Cichorioideae	Cichorieae	*Taraxacum*	*kok-saghyz*	2582325776	21388100/0.84
Cichorioideae	Vernonieae	*Centrapalus*	*pauciflorus*	3125365235	19627573/0.63
Cichorioideae	Senecioneae	*Senecio*	*vulgaris*	2045909989	15732065/0.78
Cichorioideae	Gnaphalieae	*Pseudognaphalium*	*obtusifolium*	2920317131	13066952/0.45
Asteroideae	Eupatorieae	*Conoclinium*	*coelestinum*	1746269472	20943700/1.21
Asteroideae	Heliantheae	*Phoebanthus*	*tenuifolius*	4267295897	148630586/3.52
Asteroideae	Heliantheae	*Helianthus*	*porteri*	4330738740	18192388/0.42
Asteroideae	Heliantheae	*Helianthus*	*verticillatus*	2278002736	18560744/0.82
Asteroideae	Heliantheae	*Helianthus*	*niveus ssp. tephrodes*	4192677026	23420666/0.56
Asteroideae	Heliantheae	*Helianthus*	*argophyllus*	4174346891	25568942/0.62
Asteroideae	Heliantheae	*Helianthus*	*annuus*	3384161947	22621880/0.68

[1]base pairs
