# Supplementary material for: Evolutionary transitions in the Asteraceae coincide with marked shifts in transposable element abundance
Source: BMC Genomics. 2015 Aug 20;16(1):623. doi: 10.1186/s12864-015-1830-8 (PMC4546089; doi:10.1186/s12864-015-1830-8)
Supplement: Additional file 8: — Shows published genome size estimates and genome size observations determined by the method described in this study. (PDF 54 kb) [file 12864_2015_1830_MOESM8_ESM.pdf]

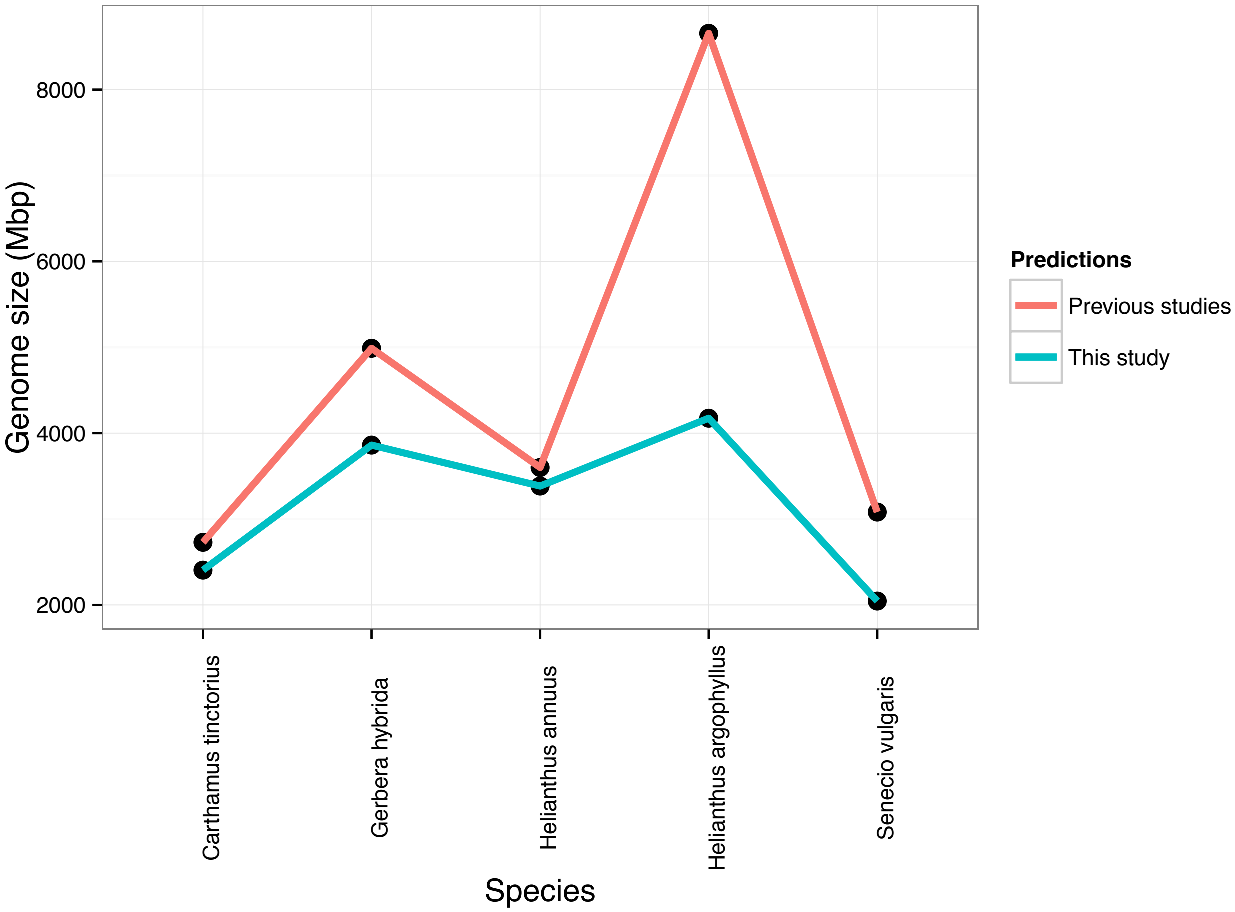

Additional file 8. Published genome size estimates and genome size observations determined by the method described in this study. Along the x-axis are species for which published genome size estimates were available (obtained from: http://data.kew.org/cvalues). The y-axis shows genome size in mega-base pairs.
